# ‘An Apple a Day’?: Psychiatrists, Psychologists and Psychotherapists Report Poor Literacy for Nutritional Medicine: International Survey Spanning 52 Countries

**DOI:** 10.3390/nu13030822

**Published:** 2021-03-02

**Authors:** Sabrina Mörkl, Linda Stell, Diana V. Buhai, Melanie Schweinzer, Jolana Wagner-Skacel, Christian Vajda, Sonja Lackner, Susanne A. Bengesser, Theresa Lahousen, Annamaria Painold, Andreas Oberascher, Josef M. Tatschl, Matthäus Fellinger, Annabel Müller-Stierlin, Ana C. Serban, Joseph Ben-Sheetrit, Ana-Marija Vejnovic, Mary I. Butler, Vicent Balanzá-Martínez, Nikola Zaja, Polona Rus-Prelog, Robertas Strumila, Scott B. Teasdale, Eva Z. Reininghaus, Sandra J. Holasek

**Affiliations:** 1 Department of Psychiatry and Psychotherapeutic Medicine, Medical University of Graz, 8036 Graz, Austria; sabrina.moerkl@medunigraz.at (S.M.); linda.stell@stud.medunigraz.at (L.S.); theresa.lahousen@medunigraz.at (T.L.); annamaria.painold@medunigraz.at (A.P.); eva.reininghaus@medunigraz.at (E.Z.R.); 2 Iuliu Hațieganu University of Medicine and Pharmacy, Faculty of Medicine, 400000 Cluj-Napoca, Romania; buhaidiana@gmail.com; 3 Department of Medical Psychology and Psychotherapy, Medical University of Graz, 8036 Graz, Austria; melanie.schweinzer@medunigraz.at (M.S.); jolana.wagner-skacel@medunigraz.at (J.W.-S.); christian.vajda@medunigraz.at (C.V.); 4 Otto Loewi Research Center (for Vascular Biology, Immunology and Inflammation), Division of Immunology and Pathophysiology, Medical University of Graz, 8036 Graz, Austria; sonja.lackner@medunigraz.at (S.L.); sandra.holasek@medunigraz.at (S.J.H.); 5 Department of Psychotherapy and Psychosomatics, University Clinic for Psychiatry, Christian-Doppler-Klinik, 5020 Salzburg, Austria; a.oberascher@salk.at; 6 Health Psychology Unit, Institute of Psychology, University of Graz, 8010 Graz, Austria; josef.tatschl@uni-graz.at; 7 Department of Psychiatry and Psychotherapy, Clinical Division of Social Psychiatry, Medical University of Vienna, 1090 Vienna, Austria; matthaeus.fellinger@meduniwien.ac.at; 8 Department of Psychiatry and Psychotherapy II, Ulm University, 89070 Ulm, Germany; annabel.mueller-stierlin@uni-ulm.de; 9 Psychiatrist in Private Sector, Psychotherapist in Cognitive Behavioural Therapy, Independent Researcher, No 26-28 Dumitru Sergiu street, sector 1, 011026 Bucharest, Romania; ana_serban1990@yahoo.com; 10 Psychiatrist in private practice, 3HaNechoshet St., Tel-Aviv 6971068, Israel; joseph.ben.sheetrit@gmail.com; 11 Department of Psychiatry and Psychological Medicine, Faculty of Medicine, University of Novi Sad, 21137 Novi Sad, Serbia; ana-marija.vejnovic@mf.uns.ac.rs; 12 Clinic of Psychiatry, Clinical Center of Vojvodina, 21000 Novi Sad, Serbia; 13 Department of Psychiatry and Clinical Neuroscience, University College Cork, T12YT20 Cork, Ireland; mary.butler@ucc.ie; 14 Teaching Unit of Psychiatry and Psychological Medicine, Department of Medicine, University of Valencia, CIBERSAM, 46010 Valencia, Spain; vicente.balanza@uv.es; 15 University Psychiatric Hospital Vrapče, University of Zagreb School of Medicine, 10000 Zagreb, Croatia; zaja.nikola@gmail.com; 16 Center for Clinical Psychiatry, University Psychiatric Clinic Ljubljana, 1260 Ljubljana, Slovenia; ruspolona@gmail.com; 17 Clinic of Psychiatry, Institute of Clinical Medicine, Faculty of Medicine, Vilnius University, 03101 Vilnius, Lithuania; robertas.strumila@gmail.com; 18 Department of Psychiatric Emergency and Acute Care, Lapeyronie Hospital, University of Montpellier, INSERM, CHU de Montpellier, 34295 Montpellier, France; 19 School of Psychiatry, UNSW Sydney, NSW 2052, Australia; scottbteasdale@gmail.com

**Keywords:** nutritional psychiatry, mental health professionals, psychiatrists, psychologists, psychotherapists, education, psychiatric disorders, diet, supplements, nutrition

## Abstract

Nutritional interventions have beneficial effects on certain psychiatric disorder symptomatology and common physical health comorbidities. However, studies evaluating nutritional literacy in mental health professionals (MHP) are scarce. This study aimed to assess the across 52 countries. Surveys were distributed via colleagues and professional societies. Data were collected regarding self-reported general nutrition knowledge, nutrition education, learning opportunities, and the tendency to recommend food supplements or prescribe specific diets in clinical practice. In total, 1056 subjects participated in the study: 354 psychiatrists, 511 psychologists, 44 psychotherapists, and 147 MHPs in-training. All participants believed the diet quality of individuals with mental disorders was poorer compared to the general population (*p* < 0.001). The majority of the psychiatrists (74.2%) and psychologists (66.3%) reported having no training in nutrition. Nevertheless, many of them used nutrition approaches, with 58.6% recommending supplements and 43.8% recommending specific diet strategies to their patients. Only 0.8% of participants rated their education regarding nutrition as ‘very good.’ Almost all (92.9%) stated they would like to expand their knowledge regarding ‘Nutritional Psychiatry.’ There is an urgent need to integrate nutrition education into MHP training, ideally in collaboration with nutrition experts to achieve best practice care.

## 1. Introduction

People with psychiatric disorders frequently experience a decreased quality of life due to disability, cormorbidity, and stigma, and have a reduced life expectancy compared to the general population [1,2]. Psychiatric disorders are significant contributors to the global burden of disease and pose one of the most pressing current challenges [3]. Traditional treatment and management strategies for psychiatric disorders have suboptimal effectiveness, and typically focus on trying to lower symptomatology, meaning disorders are often persistant rather than transient. Furthermore, individuals living with psychiatric disorders have a 15-year reduced life expectancy compared to the general population [4], predominantly driven by high rates of cardiovascular disease, diabetes, and metabolic syndrome [5]. Research on novel preventative and treatment strategies is of fundamental importance to reduce the burden of disease associated with the psychiatric disorder and common chronic disease comorbidities.

Nutritional psychiatry (NP) is an emerging field, with promising research suggesting a role of adjunctive nutritional approaches for the prevention and treatment of numerous neuropsychiatric disorders [6]. The notion that the availability of micro- and macronutrients is fundamental to brain development and function is well-established. More recently, evidence has highlighted the critical role dietary composition plays in influencing gut microbiota, neurotransmitters, neuropeptides, and the immune system, all of which are involved in the pathogenesis of psychiatric disorders [7,8,9]. Poor nutrition is considered a modifiable risk factor for general mental health and certain mental disorders. For example, eating five serves of vegetables and fruit a day is associated with better general and mental health (increased optimism and self-efficacy and lower psychological distress and depressive symptoms) [10]. This relationship between dietary intake and mental health was strengthened by a recent meta-analysis of 16 randomized controlled trials (RCTs; *n =* 45,826), which found that nutritional interventions significantly reduced depressive symptoms, particularly when they were delivered by accredited nutritional professionals (e.g., dietitians or nutritionists) [11]. The effects of nutrition interventions are comparable to behavioral therapy and superior to a ‘social support group’ in patients suffering from depression [12,13]. Given the inadequacies of traditional preventative, treatment, and management strategies in psychiatry when used alone, greater importance should be placed on adjunctive strategies such as nutritional psychiatry [14,15].

Moreover, individuals with mental health problems frequently have an unhealthy lifestyle including poor dietary choices, disordered eating behaviours, and nutritional deficiencies [16,17]. This is driving, in part, the high rates of chronic disease and reduced life expectancy [2]. Therefore, lifestyle modification (including diet) should be incorporated as best-practice management of physical comorbidities in people with psychiatric disorders [18]. It is reassuring to note that the nutrition recommendations and dietary patterns for protecting peoples’ physical health are in line with those which are beneficial for mental health.

It is critical that mental health professionals (MHPs) have basic training and literacy in nutrition so they can provide preliminary nutrition advice to patients and refer them to nutrition experts as necessary. However, the training, literacy level and use of nutrition approaches by key MHPs, psychologists and psychiatrists, remains unclear. European and US studies have investigated current nutrition training in general medical curricula and found that nutritional medicine is either not taught, or insufficiently taught, in medical school [19,20,21]; however, there are some recent developments to integrate nutrition in medical education (e.g., PAN-int.org). Additionally, some universities have begun offering certifications in nutritional psychology, but there are no official regulations or standards.

To our knowledge, there is a paucity of studies on the perception, education, and awareness of psychiatrists, psychologists, and psychotherapists in relation to nutrition literacy and interventions. One small study (*n* = 6) investigated subjective opinions of psychotherapists regarding nutrition [22] and suggested that dietary issues should become more integrated into the field of psychotherapy. In a survey from 1989, 232 American psychologists reported that they received no education regarding nutrition, but more than half of the participants believed that diet and exercise should be a mandatory component in the graduate school curriculum [23]. No newer studies on this subject were identified by the authors. Based on the existing curricula, a large educational gap regarding nutritional education is still foreseeable.

Thus, we formulated the following hypotheses: (1) MHPs globally have engaged in little-to-no nutrition education and self-perceived nutrition literacy is low, as nutrition approaches are not taught in graduate or postgraduate MHP courses, (2) nutrition care is not integrated in routine clinical practice as nutritional literacy is low, and (3) nutrition interventions (i.e., diet or supplements) are not applied in clinical practice. The aim of this study was to investigate the level of nutrition education taught in university programs, self-perceived nutrition literacy, and use of nutritional approaches (diet and/or supplements) in clinical practice.

## 2. Materials and Methods

### 2.1. Recruitment of Participants and Group Characteristics

The online survey was approved by the ethics committee of the Medical University of Graz, Austria (No: EK 31-021ex 18/19). Data were acquired using an anonymous, self-rated questionnaire accessible via Google forms (https://www.google.com/forms/about/, accessed on 12 December 2018). The questionnaire was distributed via email to national and international colleagues using a combined snowball sampling approach. All psychiatrists of Austria received an invitation to participate via the Austrian medical chamber. Psychiatrists and psychologists worldwide were contacted through the early career psychiatrists (ECP) network of the World Psychiatric Association (WPA) and local professional psychiatric associations.

Participants provided informed consent electronically before participation. The informed consent page presented two options (‘yes’/’no’). Only subjects who chose ‘yes’ were taken to the questionnaire page, and participants could end their participation at any time.

Inclusion criteria were as follows: (i) psychologists, psychiatrists, and psychotherapists as well as psychologists in training and psychiatrists in training (psychiatry registrars), (ii) aged between 24 and 100 years, (iii) gave their informed consent to take part in the online survey, and (iv) ability to sufficiently understand the online questionnaire (which was available in the English and German languages). Exclusion criteria were: (i) aged <24 years, since 24 is generally the minimum age to finish medical school/university and start a career in psychiatry or clinical psychology, and (ii) medical doctors in other specialties.

### 2.2. The Online Survey

The survey was developed by a consensus approach between the lead authors. Participants received a standard, non-personalized online link via email, granting them access to the content of the survey. The survey could be completed via a computer, tablet, or smartphone and took approximately 10–15 min to complete. No pre-registration was required from the participants. Following the consent page, the survey consisted of: demographical data, perceived importance of nutrition, self-rated knowledge, formal education, and current use in clinical practice. Questions were answered on a 10-point Likert scale from 1 (very low) to 10 (very high). A 10-point Likert scale was used, as it offers more variance than the traditional five-point Likert scales, as well as higher measurement precision and greater opportunity to detect subtle differences [24]. Survey questions and answers can be accessed in the supplementary files (Supplementary Files S1 and S2).

### 2.3. Statistical Evaluation and Data Management

The analyses were conducted in SPSS v23.0 (IBM, Armonk, New York, NY, USA). Data visualization was performed using Microsoft Excel 365 (Microsoft Corporation, Redmond, USA). Unless stated otherwise, descriptive results of continuous variables are expressed as mean and standard deviation (SD) for Gaussian distributed variables. Data of the Likert scales are expressed as Median (Mdn) and Interquartile Range (IQR). Depending on the distribution of data and type of data, we performed an ANOVA, Mann-Whitney U test, Kruskal-Wallis-Test, or χ^2^-test to identify differences between professional groups. Professional subgroup analyses were conducted for demographical data (sex, age, working experience), current perceptions of ‘Nutritional Psychiatry’, and current treatment practices. Correlations between variables (current rated knowledge, work experience, rated importance of ‘Nutritional psychiatry’, and rated ‘importance of discussion nutrition with patients’) were calculated with Spearman’s correlation coefficient. Unless otherwise specified, missing survey data were mostly treated as user-defined missing values in SPSS. Data were screened for plausibility and manually converted into missing values if they were not plausible. Results for post-hoc tests were corrected with the Bonferroni correction for multiple comparisons. Levels of statistical significance were set at *p* < 0.05 (two-tailed).

## 3. Results

**Figure 1:**
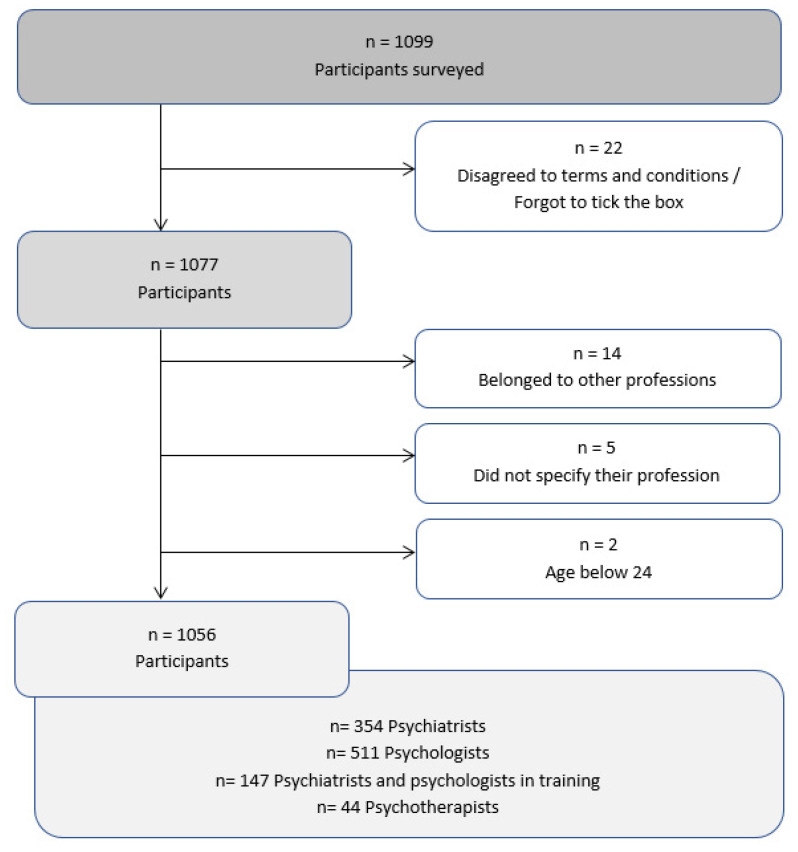
Overview of participants surveyed.

Data were collected for a total of 22 months (from December 2018 to September 2020). Participants who did not agree to the terms and conditions (*n* = 22) and those who did not meet the inclusion criteria (*n* = 21) were excluded from the analysis. The remaining participants (*n* = 1056) were included in the data analysis. When someone had several professions (for example being a psychiatrist and a psychotherapist), the reported main profession (psychiatrist) was counted. Figure 1 gives an overview of the flow of participants through the study.

### 3.1. Demographical Data of Study Participants

A final sample of 1056 participants, 354 psychiatrists, 511 psychologists, 147 psychiatry and psychology trainees, and 44 psychotherapists, from 52 countries, were included. The majority of the participants were female (71.9%), and the mean age was 39.9 (SD 10.0) years.

The majority of participants reported working in a hospital (*n* = 450, 42.6%), followed by private practice (*n* = 233, 22.1%), mental health outpatient services (*n* = 178, 16.9%), rehabilitations centres (*n* = 64, 6.1%), and day clinic (*n* = 20, 1.9%). A further 10% reported working in institutions other than those listed above, and one participant did not specify their place of work.

Table 1 gives an overview of the participant’s country of occupation, grouped by country income level. Of those who reported their country (*n* = 1047), most were based in high-income countries (*n* = 905, 86.4%), followed by upper-middle income countries (*n* = 121, 11.6%), lower-middle income countries (*n* = 20, 1.9%), and only 1 (0.1%) from a low income country. By region, most participants were in Europe (31 countries, *n* = 866, 82.7%), followed by Asia (10 countries, *n* = 108, 10.3%), North America (3 countries, *n* = 34, 3.2%), Oceania (1 country: Australia, *n* = 19, 1.8%), South America (3 countries, *n* = 12, 1.1%), and Africa (4 countries, *n* = 8, 0.8%).

**Figure 2:**
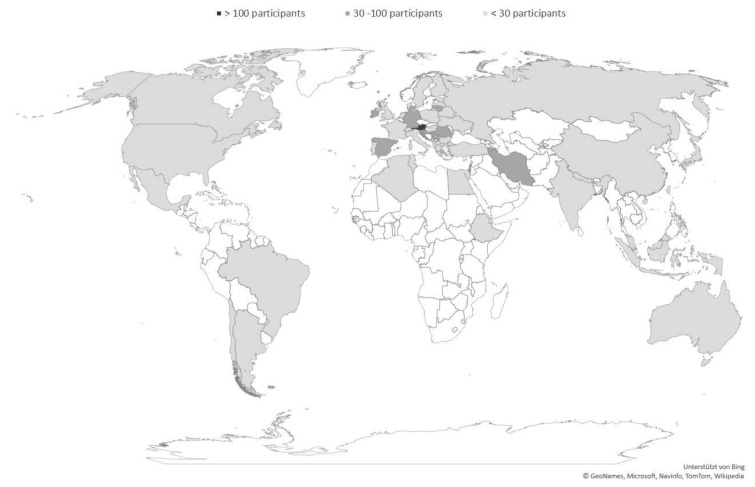
World map showing all participating countries. Light gray color indicates countries with few participants; dark gray color indicates countries with numerous participants. This map was created with Microsoft Excel^®^ (own figure).

Countries with the largest participation were: Austria (*n* = 481), Romania (*n* = 54), Israel (*n* = 52), Germany (*n* = 40), and Serbia (*n* = 35). Figure 2 illustrates the degree of participation by country.

Participants were specialized in general adult psychiatry or psychology (*n* = 467, 44.2%), child and adolescent psychiatry or psychology (*n* = 151, 14.3%), neuropsychiatry or neuropsychology (*n* = 54, 5.1%), psychosomatics (*n* = 49, 4.6%), psychogeriatrics (*n* = 36, 3.4%), addiction medicine (*n* = 22, 2.1%), and forensic psychiatry or psychology (*n* = 15, 1.4%). According to 72 (6.8%) participants, their specialization was not listed, a further 154 (14.6%) reported to have no specialization, and 35 (3.4%) gave no answer.

Table 2 gives an overview of participants and comparisons of the main characteristics of psychiatrists, psychologists, and psychotherapists.

Sex differed significantly across all groups (χ^2^ (6, *N* = 1054) = 100.0, *p* < 0.001). While approximately half of the psychiatrists were male, psychologists and psychotherapists were predominantly female (Table 2). Furthermore, groups differed significantly regarding age (H (3) = 172.07, *p* < 0.001), with psychologists being slightly older than both psychiatrists and psychotherapists (for both, *p* < 0.05). As expected, psychiatrists and psychologists were significantly older than those in training (*p* < 0.001). Additionally, a significant difference in the work experience between the groups was found (H (3) = 18.185, *p* < 0.001). There was a significant difference in the duration of working experience between participants in training and all other groups: psychologists and participants in training (*p* < 0.001), psychiatrists and participants in training (*p* = 0.012), and psychotherapists and participants in training (*p* = 0.011).

In relation to the year of postgraduate education, 304 (28.8%) participants had specified their current year of postgraduate education, 561 (53.2%) participants had completed their studies, and 191 (18.2%) did not specify.

### 3.2. Nutritional Education

Of the 511 psychologist participants, 51.1% (*n* = 261) responded as to whether they received nutrition education during postrgraduate studies: among the responding psychologists, 173 (66.3%) reported no lectures, 59 (22.6%) reported some training during their studies in psychology, 22 (8.4%) attended electives on this topic, and 7 (2.7%) had obligatory courses (Austria *n* = 6 and Germany *n* = 1). Of the 354 psychiatrist participants, 198 (55.9%) psychiatrists responded to the question of whether they had specific training in the nutritional care of patients during their psychiatric residency: 147 (74.2%) psychiatrists reported no lectures, 30 (15.2%) had some training during their diploma of medicine, and 17 (8.6%) completed electives on this topic. A minority of participants (*n* = 4, 2.0%) had obligatory courses (Lithuania *n* = 1, Switzerland *n* = 1, UK *n* = 1 and USA *n* = 1).

While working as a psychiatrist or psychologist, 111 (10.5%) participants had attended specific training in nutritional care. Regarding awareness of courses that teach nutrition, 229 (21.7%) participants were aware of courses that teach nutrition for prevention and treatment of psychiatric disorders in their country or at their institution: 94 (41.1%) psychologists, 80 (34.9%) psychiatrists, 45 (19.7%) in training, and 10 (4.4%) psychotherapists.

Nearly all participants (92.9%) would be willing to expand their knowledge of ‘Nutritional Psychiatry.’ The most popular avenue of learning was ‘congresses’ (*n* = 650), followed by ‘scientific journals’ (*n* = 495) and ‘interdisciplinary meetings’ (*n* = 480). The least popular avenue was engaging in ‘Master studies’ (*n* = 80) and ‘Ph.D. projects’ (*n* = 59).

Perceived current knowledge in ‘Nutritional Psychiatry’correlated positively with (i) participants perceived ability to improve their quality of work and participant outcomes by training in ‘Nutritional Psychiatry’ (*r*_s_ = 0.329, *p* < 0.001), (ii) the rating of the importance of ‘Nutritional Psychiatry’ (*r*_s_ = 0.393, *p* < 0.001), and (iii) the rating for importance of discussing nutrition with patients (*r*_s_ = 0.396, *p* < 0.001).

### 3.3. Treatment Practices

Two-hundred and thirty eight (67.2%) psychiatrists, 335 (65.6%) psychologists, and 29 (65.9%) psychotherapists reported using nutritional approaches for the treatment of patients, with no significant differences between the professional groups (χ^2^ (3, *N* = 1056) = 0.556, *p* = 0.906).

Nutritional approaches were most frequently used for the treatment of eating disorders (*n* = 436 answers) and affective disorders (*n* = 344 answers), followed by anxiety disorders (*n* = 208 answers), psychotic disorders (*n* = 130 answers), and obsessive-compulsive disorders (*n* = 58 answers). One-third (*n* = 379 (35.9%) of the participants reported having never used a nutritional approach for any psychiatric disorder. For the prevention of somatic comorbidities, 402 participants (38.1%) reported using nutritional interventions occasionally, while 212 (20.1%) reported never using such interventions, and only 43 participants (4.1%) always included nutritional interventions.

Nearly a quarter of the participating psychiatrists (*n* = 88; 24.9%) reported considering the individual nutritional status of patients when prescribing psychopharmacological therapy intermittently, 67 (18.9%), reported considering it most of the times, and 22 (6.2%) reported considering it always. Eighty-three (23.4%) reported to never and 62 participants (17.5%) to hardly ever consider the nutritional status of patients when prescribing psychopharmacological medication.

The most recommended lifestyle intervention was physical activity (*n* = 935), followed by dietary coaching (*n* = 558) and cooking classes (*n* = 112), while 102 participants reported hardly ever recommending lifestyle intervention. Most participants reported never (*n* = 498, 47.2%) or hardly ever (*n* = 306, 29.0%) testing for food allergies, gluten sensitivity, or food intolerances. There was no difference between the professional groups (χ^2^ (12, *N* = 1009) = 8.058, *p* = 0.781) in testing for food allergies, gluten sensitivity, or food interolerances.

Nutritional care in the context of mental disorders was considered as ‘very important’ by 121 (11.4%) of the participants (Likert scale 10/10). Discussing nutrition in the clinical setting was rated as very important (Likert scale 10/10) by 73 (6.91 %) of the participants.

When asked about rating the nutrition of the countries’ population, the most frequent rating was 5/10 (*n* = 199, 18.8%); only 6 (0.6%) rated the status as “very good” (Likert scale 10/10). When asked about the nutritional status of individuals with mental disorders in their country, the most frequent rating was 3/10 (*n* = 294, 27.8%); only 1 person (0.1%) rated the quality with ‘very good.’ Importantly, participants rated the dietary quality of individuals with mental disorders (Mdn = 3.00) as significantly worse when compared to the general population (Mdn = 5.00) of their countries (U = 265739.00, *p* < 0.0001). Participants most frequently rated the quality of food served in hospitals within their country of occupation as 5/10 (*n* = 199, 18.8%). There was no significant difference between the qualification groups (*H*(3) = 1.841, *p* = 0.606).

Regarding regular screening for comorbid metabolic disorders, 314 (*n* = 29.7%) were aware of regular screening in their place of work, whereas nearly half of the participants did not know or provided no answer (*n* = 469; 44.4%) and 264 (25.0%) were unaware of regular screening. Interestingly, there was a significant difference between the professions (χ^2^ (6, *n* = 1047) = 22.31, *p* = 0.001). After a Bonferroni correction for multiple testing, psychiatrists (*p* = 0.006) and psychologists (*p* = 0.006), as well as psychotherapists, were significantly more aware of metabolic screening in their countries compared to those in training.

### 3.4. Recommendation of Diets and Dietary Supplements by MHPs

Nearly half (*n* = 462; 43.8%) of participants have recommended a specific diet to their patients. Participants could tick one or multiple boxes and had to choose from the following diets: diet in accordance with national guidelines, Mediterranean diet, vegetarian diet, vegan diet, ketogenic diet, low carb diet, Glyx diet, and/or other diets. The most recommended diets were a Mediterranean diet (*n* = 210) and a diet in accordance with national guidelines (*n* = 202), followed by a low-carb diet (*n* = 135) and others (*n* = 104). The Glyx diet (*n* = 15) and vegan diet (*n* = 17) were the least recommended. Table 3 and Table 4 provide an overview of other diets that could be given in a free text answer field. When asked to specify the indications for recommending the diet (multiple answers were possible), most of the participants named ‘metabolic comorbidities’ (*n* = 421), followed by ‘prevention of adiposity’ (*n* = 387) and ‘obesity’ (*n* = 330). For psychiatric indications, ‘Eating disorders’ (*n* = 337) was ranked first, followed by ‘symptoms of depression’ (*n* = 282), ‘symptoms of anxiety’ (*n* = 159), and ADHD (*n* = 140). Regarding recommendation of a specific diet to patients, there were no significant differences between male and female MHPs (χ^2^ (2, *N* = 1034) = 3.246, *p* = 0.197). Almost half of the participants (*n* = 520, 49.2%) reported having already started a diet themselves and maintained it for at least one month. There was no significant difference between professional groups (χ^2^ (3, *N* = 1036) = 3.904, *p* = 0.272) and male and female MHPs (χ^2^ (2, *N* = 1033) = 1.016, *p* = 0.602).

Next, we asked all participants whether they recommended nutritional supplements to their patients. A total of 619 (58.6%) participants reported recommending nutritional supplements: 64.5% of psychologists (*n* = 323), 57.2% of psychiatrists (*n* = 198), 54.5% of psychotherapists (*n* = 24), and 51.0% of psychologists and psychiatrists in training (*n* = 74). There was a significant difference between professions (χ^2^ (3, *n* = 1036) = 10.635, *p* = 0.014). Psychologists recommended more supplements than psychotherapists (χ^2^ (1, *n* = 397) = 8.571, *p* = 0.003), of which the results were statistically significant after Bonferroni correction (*p* = 0.018). The higher rates of psychologists recommending supplements compared to psychiatrists (χ^2^ (1, *n* = 521) = 4.538, *p* = 0.033) was not statistically significant after Bonferroni correction for multiple testing *(p* = 0.198). There were no significant sex differences regarding the recommendation of supplements (χ^2^ (2, *N* = 1033) = 3.758, *p* = 0.153).

We provided a list of commonly recommended supplements for mental health which could be ticked in the instance participants ever recommended them to their patients (vitamin D, omega-3, vitamin A, vitamin E, selenium, zinc, magnesium, vitamin B6, vitamin B12, folic acid, iron, N-acetylcysteine). For this question, it was possible to tick more than one box. The most recommended supplement was vitamin D (*n* = 446), followed by vitamin B12 (*n* = 414), omega-3 (*n* = 364), folic acid (*n* = 319), and vitamin B6 (*n* = 314).

An additional 164 participants (15.5%) reported recommending supplements not listed in the survey or gave additional answers. Table 5 lists the additional supplements recommended by participants.

Most participants (*n* = 853, 79.1%) reported taking, or having taken, supplements themselves, while 206 participants (19.5%) reported never having taken supplements, and 15 (1.4%) gave no answer. There was no significant difference between professions (χ^2^ (3, *N* = 1041) = 5.384, *p* = 0.146) or gender (χ^2^ (2, *N* = 1038) = 3.504, *p* = 0.173) for supplement intake.

Table 6 lists the additional comments the participants gave when asked about recommended supplements.

Regarding probiotics, 328 participants (31.1%) reported recommending probiotics to their patients, with no significant between profession difference (χ^2^ (3, *N* = 1035) = 3.410, *p* = 0.333).

## 4. Discussion

In this international, cross-sectional survey, we investigated subjective nutritional literacy, nutritional education, and the use of nutritional interventions (such as diets and supplements) in 1056 MHPs from 52 countries. MHPs consider nutrition as an important pillar in the biopsychosocial care model. However, most of the MHPs reported having little or no nutrition literacy and no professional training in nutrition; nevertheless, nutritional approaches were being recommended by half of the MHPs, and 60% of these recommendations were for the treatment of psychiatric disorders. It appears likely that these nutritional approaches are being recommended without an adequate knowledge base.

### 4.1. Education

The limited nutrition discussion and education from MHPs to patients may be due to an inadequate education and subsequently limited confidence in advising patients [23]. Nutritional medicine is not adequately taught in medical schools irrespective of future speciality; for example, only 40% of US medical schools reach the goal of teaching 25 h of nutrition in preclinical years [20,21]. This education gap appears to be present worldwide. An assessment of medical nutrition education in 15 European countries and six non-European countries concluded that ‘nutrition is insufficiently incorporated into medical education, regardless of country, setting, or year of medical education’ [21]. This was reflected in our findings; more than two-thirds of psychiatrists and psychologists reported that they had no specific training in nutrition, with only a minority (2.68% of psychologists and 2.02% of psychiatrists) who undertook mandatory courses.

We hypothesized that psychiatrists may rate their knowledge concerning nutrition significantly higher compared to the other professional groups due to their medical training. However, there was no significant difference of nutritional literacy between psychiatrists and the other professional groups. Importantly, therapists are more likely to advocate for healthy habits when they have adequate knowledge and are practicing a healthy lifestyle themselves [107]. Moreover, an American study found that 63% of psychiatric healthcare providers practiced poor nutritional habits, though they considered themselves as role models to patients [108].

Hence, in conjunction with the current literature, our findings emphasize the need to implement targeted nutritional education for MHPs. Importantly, we suggest that undergraduate/graduate curriculums incorporate mandatory nutritional education, as the majority of MHPs seem reluctant to participate in post-graduate training. Additionally, congresses, journal articles, and interdisciplinary meetings could be feasible tools to foster interest in the field of “Nutrional Psychiatry,” as these approaches were the most popular among the participants of this study.

The US Academy of Nutrition and Dietetics recommends that registered dieticians should play a significant role in the interprofessional education of medical students, residents, and physicians in practice [109]. This interprofessional approach should also be applied to the education of MHPs, the majority of which (90%) would like to expand their knowledge in ‘Nutritional Psychiatry.’ Based on our findings, the current practice of nutrition therapy in clinical psychiatry is untenable from an evidence-based medicine perspective with MHPs recommending questionable diets and supplements despite having little to no training in nutrition therapy.

Some psychologists answered that they would refer their patients to medical doctors (e.g., a psychologist wrote ‘*I send my clients to see a doctor if they ask me about nutrition*’). Sending patients to see a psychiatrist or a medical doctor of another specialty may not be the best advice, as nutrition does not seem to be a mandatory subject in medical curricula in many countries. Additionally, the lack of reported referrals to nutrition specialists appears to be a gap in collaborative care and likely impeding best patient outcomes.

### 4.2. Treatment Practices

In our study, all professional groups used nutritional approaches for the treatment of psychiatric disorders despite not having education in nutritional medicine, with eating disorders and affective disorders being the most prominent indications. For somatic comorbidities, more than one-third of the participants reported using nutritional interventions occasionally. This lack of education of MHPs may be the reason why dietary interventions significantly reduced depressive symptoms in a meta-analysis; however, only when delivered by accredited nutritional professionals (e.g., dietitians or nutritionists) [11].

As psychopharmacological medication can cause severe metabolic consequences, nutritional approaches could be an ideal adjunctive treatment. However, only 6.2% of the psychiatrists in our survey reported always considering the nutritional status of patients when prescribing psychopharmacological therapy and half of the participants were not aware of any regular screening for metabolic disorders in psychiatric paitents in their country. This is also reflected by studies on this topic: although metabolic syndrome is common in patients taking psychopharmacological medication, hardly any patient has regular metabolic screening [110,111].

Additionally, physical health is neglected by most of the patients themselves and goes hand in hand with an increased prevalence of somatic illness such as obesity, diabetes, and cardiovascular diseases, followed by a significant reduction in life expectancy of 10–20 years in comparison to the general population [112]. Importantly, our survey participants rated the dietary quality of individuals with mental disorders as significantly worse when compared to the general population of their countries.

Therefore, treatment practices have to be improved to contain nutritional advice for patients, complementing other recommended lifestyle interventions such as physical activity.

### 4.3. Recommended Diets and Supplements by MHPs

Studies have revealed that certain dietary types, such as the Mediterranean diet, are associated with a lower incidence of depression [113,114,115] and that diet represents a major factor shaping the gut microbiome and its metabolites. Nearly half of our survey participants (43.8%) reported recommending special diets for patients with psychiatric disorders, with the Mediterranean diet being the most popular choice. A Mediterranean diet ensures an adequate nutritional intake [116], combines the beneficial effects of single nutrients, and targets a variety of mechanisms including anti-inflammatory, antioxidant, neurogenesis, and microbiome- and immune-modifying activities [117]. For example, the large European PREDIMED study demonstrated a reduced risk for incident depression in people with type 2 diabetes who were randomized to a Mediterranean diet supplemented with nuts, compared with a low-fat diet control group [118]. Conversely, a vegan diet was the least recommended diet by the survey participants. Indeed, according to a recent systematic review, vegan or vegetarian diets were found to be related to higher risks of depression but lower levels of anxiety [119]. A striking finding of our study was the remarkable number of various additional diets for mental health recommended in a free text answer field by MHPs (see Table 3). While for some diets there is evidence for their beneficial effects, others are not sufficiently researched and their potential harmful effects for patients cannot be entirely ruled out based on current evidence.

Even more participants recommended supplements instead of a special diet for patients with psychiatric disorders (58.6% vs. 43.8%). Recent study results indicate that untargeted supplementation of nutraceuticals (of both single vitamins or multi-vitamins or minerals) may not be equal to the recommendation of a properly balanced diet such as the Mediterranean diet providing food products [86,120,121,122]. The most recommended supplements in our survey were vitamin D, vitamin B12, and omega-3-fatty acids. While there is some evidence for supplementing these nutrients in psychiatric disorders [123,124,125,126], 164 (15.5%) participants reported recommending a range of additional supplements. Indeed, nutraceuticals seem to be widely used for the treatment of psychiatric disorders. The nutraceutical market is a quarter of the global pharmaceutical market, with growth potential in the years to come. Patients with psychiatric disorders frequently take supplements [127,128], because some 40% of patients may not satisfactorily respond to antidepressant drugs [129] and approximately 50% of psychiatric patients prematurely discontinue their psychopharmacological treatment due to side effects [130]. A substantial proportion of patients do not reach complete remission with state-of-the-art therapies, which reflects our incomplete understanding of the complex etiology and pathophysiology of most psychiatric disorders [131]. We assume that MHPs may recommend supplements in an effort to improve a potential unsatisfactorily treatment response of their patients or the frequent demand of patients to find a suitable, ‘natural’, ‘complementary’, or ‘alternative’ treatment with an estimated lower incidence of side effects [132]. Hence, there is an urgent need to complement the current treatment paradigm with safe and sustainable interventions. Without question, micronutrients are vital to neurotransmitter synthesis and proper functioning of the nervous and the immune system. Several micronutrients, such as selenium, zinc, iron, magnesium, vitamin B12, and folic acid, were found to be inversely associated with increased depression risk [7,8,9,133,134] and some nutraceuticals, such as 5-hydroxytryptophan, omega-3 fatty acids, or folic acid, are used as adjunctive treatments in psychiatry [125,135,136].

However, for most supplements, the efficacy for psychiatric indications is not sufficiently researched and evidence-based recommendations are lacking for many [121]. While some supplements have been used in traditional medicine systems for thousands of years, there is a paucity of high-grade evidence for most of the supplements recommended by MHPs for the treatment of psychiatric disorders (see Table 5). Some of these supplements may have mechanisms of actions on the central nervous system as well as the gut–brain axis that are yet to be discovered, and more research is necessary. Additionally, long-term effects and side effects for most of the reported supplements are unknown. In many countries, supplements are regulated as food and not as pharmaceutical drugs which require a prescription. In our survey, psychologists reported recommending significantly more supplements than psychiatrists and psychotherapists, although the significant difference between psychologists and psychiatrists did not remain significant after correction for multiple comparisons. We suspect that psychologists may recommend supplements as an aid for therapy, because they are, in most countries, by law not allowed to prescribe medication. Nevertheless, dietary supplements may contain ingredients exhibiting strong biological effects that may interact with psychopharmacological medication [137,138]. Therefore, medical and nutritional literacy is required to prevent adverse effects for patients.

### 4.4. Strengths and Limitations

Our present study has several strengths: to the best of our knowledge, to date, this is the first and the largest study on this topic. We have a reasonably high number of participants, covering 52 countries worldwide from all income-groups. However, some countries (such as Austria) had a very high number of participants, while the response rate was significantly lower for other countries, making direct comparisons between professionals of different countries difficult. Moreover, most of our survey participants (71.9%) were female—this is not unexpected, however, since MHPs are predominantly female [139,140]. In any case, there were no significant sex differences regarding the rated knowledge on “Nutritional Psychiatry” or recommendations of special diets or supplements for psychiatric disorders. Nevertheless, there could be a gender bias in the results, as females may have been more interested in participating in nutrition surveys as females tend to have greater interest in healthy diets and lifestyles [141]. As is always the case in online surveys, the rating of nutritional knowledge of the participants is based on subjective self-perception. Some participants in the group of psychiatrists and psychologists in training reported having more than 40 years of work experience, which results in a mean work experience of 13.9 years. This could have been due to medical doctors who have more than one specialization (for example, one participant stated that he first specialized in internal medicine, and then started his training in psychiatry later on in his career).

Another obvious limitation is the potential for ‘selection bias’, with those who have an interest in nutrition more likely to participate in such a survey. Additionally, the educational and occupational standards vary widely across the countries. Moreover, culturally established approaches may be reflected in the application of nutraceutical therapies. Given the fact that the received links were not customised, duplicate participation in the study could theoretically occur, though we feel this is unlikely to have been present to an extent that would significantly influence the results (given the time required to complete the survey and no additional benefit to the participant for completing it more than once). The survey was primariliy distributed via email to national and international colleagues using a combined snowball sampling approach. As participation of professions other than MHPs could not be ultimately ruled out due to the anonymous, self-rated nature of the survey, the questionnaire covered a question regarding current medical qualification. In case a participant reported not being a psychiatrist, psychologist, or psychotherapist, these data were excluded from the survey (as listed in Figure 1; *n* = 14 participants belonged to other professions and *n* = 5 did not specify their profession). Lastly, the snowball sampling technique is commonly used in websurveys such as the present one. The non-probabilistic nature of the sample precludes generalizability of the results to the entire population of MHPs.

### 4.5. Implications for Future Research

Future research should focus on the effectiveness and efficacy of nutrition as part of the educational curriculum for MHPs in order to sustainably integrate nutrition into the biopsychosocial treatment model and to avoid treatment errors and detrimental health effects of supplement recommendation without evidence. Most of our study participants reported that the quality and outcome of work could be improved by further training in ‘Nutritional Psychiatry’, and nearly all MHPs would like to expand their nutritional literacy.

As one of the first universities in Europe, we started a training program for medical students on nutritional medicine and mental health at the Medical University of Graz, Austria in 2018. Currently, we are investigating the effects of this training program in terms of creating awareness of the topic and utilizing this knowledge into clinical practice.

## 5. Conclusions

As a first step, this international survey aimed to create awareness of an alarmingly lack of literacy concerning nutritional medicine in MHPs despite the rapidly evolving evidene base for the use of adjunctive nutritional therapies in the routine care of psychiatric patients. Improving current educational curricula and incorporating appropriate modules on nutritional psychiatry appears critical given that mental health care costs are increasing.

Subsequently, patients should expect appropriate, evidence-based basic advice early in the course of treatment with the option to refer to nutrition specialists (medical doctors trained in nutritional medicine, nutritionists, dietologists, dietitians) as necessary. This collaborative process has the potential to improve outcomes related to the mental disorder and common metabolic comorbidities [142].

Most importantly, the medical maxim of ‘first, do no harm’ should be followed by avoiding the recommendation of supplements or diets without sufficient scientific evidence and a preceding physical examination and laboratory testing (including screening for deficiencies). The next generation of MHPs should not only be able to treat patients with state-of-the-art psychotherapy and psychopharmacology, but should also interest their patients in the care of the body and the brain, in diet, and the multifactorial cause and prevention of psychiatric disorders.

## Figures and Tables

**Table 1:** Income levels, country, and number of psychiatrists/psychologists working in the mental health sector.

Income Group (June 2020)	Country	Continent	Participants (per Country)	Psychiatrists Working in Mental Health Sector (per 100,000 Population)	Psychologists Working in Mental Health Sector (per 100,000 Population)
High income	Australia	Oceania	**19**	*13.5*	*103.0*
	Austria	Europe	**481**	20.7 *	0.2 **
	Canada	North America	**4**	*14.7*	*48.7*
	Chile	South America	**1**	*7.00*	*NA*
	Croatia	Europe	**32**	*11.1*	*4.4*
	Denmark	Europe	**2**	17 ^†^	*NA*
	Estonia	Europe	**11**	*16.2*	*6.5*
	Finland	Europe	**2**	*23.6*	*109.5*
	France	Europe	**4**	*20.9*	*48.7*
	Germany	Europe	**40**	*13.2*	*49.6*
	Greece	Europe	**2**	*5.8*	*8.8*
	Hungary	Europe	**1**	*11.1*	*2.5*
	Ireland	Europe	**33**	19.0 ***	6.0 ^†^
	Israel	Asia	**52**	*9.9*	*88.1*
	Italy	Europe	**8**	*6.0*	*3.8*
	Japan	Asia	**4**	*11.9*	*3.0*
	Latvia	Europe	**1**	*1.0*	*NA*
	Lithuania	Europe	**30**	*18.5*	*15.9*
	Malta	Europe	**1**	*NA*	*NA*
	Netherlands	Europe	**5**	*20.9*	*123.5*
	Poland	Europe	**3**	*24.2*	*16.4*
	Portugal	Europe	**2**	11^†^	*NA*
	Romania	Europe	**54**	*5.7*	*1.5*
	Slovenia	Europe	**31**	*12.0*	*9.3*
	Spain	Europe	**32**	*9.7*	*NA*
	Sweden	Europe	**3**	22^†^	66 ^†^
	Switzerland	Europe	**19**	*44.0*	*84.1*
	Taiwan	Asia	**1**	*NA*	*NA*
	UK	Europe	**15**	20^†^	16 ^†^
	USA	North America	**12**	*10.54*	*29.9*
Upper-middle income	Albania	Europe	**1**	*1.5*	*1.2*
	Argentina	South America	**2**	*21.7*	*222.6*
	Belarus	Europe	**2**	*13.5*	*5.5*
	Brazil	South America	**9**	*3.2*	*12.4*
	Bulgaria	Europe	**1**	*7.2*	*1.9*
	China	Asia	**2**	*2.2*	*NA*
	Indonesia	Asia	**2**	*0.3*	*0.2*
	Iran	Asia	**34**	*2.0*	*5.2*
	Macedonia	Europe	**2**	*14.4*	*2.4*
	Malaysia	Asia	**2**	*1.1*	*1.0*
	Mexico	North America	**18**	*0.2*	*3.5*
	Montenegro	Europe	**1**	*8.3*	*NA*
	Russia	Asia (Europe)	**7**	*8.5*	*4.6*
	Serbia	Europe	**35**	*8.6*	*4.6*
	Turkey	Europe	**3**	*1.6*	*2.5*
Lower-middle income	Algeria	Africa	**1**	*NA*	*NA*
	Egypt	Africa	**3**	*1.6*	*0.3*
	India	Asia	**3**	*0.3*	*0.1*
	Nepal	Asia	**1**	*0.4*	*0.5*
	Tunisia	Africa	**3**	*NA*	*0.01*
	Ukraine	Europe	**9**	*6.9*	*NA*
Low income	Ethiopia	Africa	**1**	*NA*	*NA*

Data given in bold are the numbers of participants from each country who participated in the survey. Data given in italics are derived from the World Health Organization (WHO): GHO|By category|Human resources—Data by country. (2019). Retrieved 23 November 2020, from https://apps.who.int/gho/data/node.main.MHHR?lang=en (accessed on 23 November 2020); * Data source: Austrian Medical Chamber, 2020 ** Data source: Federal Ministry of Austria, List of health psychologists according to § 17 Psychologengesetz 2013, BGBl. I Nr. 182/2013 and List of clinical psychologists according to § 26 Psychologengesetz 2013, BGBl. I Nr. 182/2013, from https://www.sozialministerium.at/Themen/Gesundheit/Medizin-und-Gesundheitsberufe/Berufslisten.html (accessed on 3 December 2020). *** Data source: The College of Psychiatrists of Ireland Workforce Planning Report 2013–2023 December 2013. From https://www.irishpsychiatry.ie/wp-content/uploads/2016/10/CPsychI-Workforce-Planning-Report-2013-2023-Dec-2013.pdf (accessed on 15 December 2020); ^†^ data source: OECD (2014), [25].

**Table 2:** Main characteristics of participants according to their profession.

		Psychiatrists	Psychologists	Psychotherapists	Psychiatrists and Psychologists in Training	*p*-Value
		(*n* = 354)	(*n* = 511)	(*n* = 44)	(*n* = 147)	
Sex	*n*	193	428	34	102	<0.001
(female)	*%*	54.5	84.3	77.3	69.4	
Age	mean	40.9	42.1	40.2	30.5	<0.001
(years)	standard deviation	11.1	10.5	11.5	4.6	
Working experience	mean	12.1	10.9	10.2	13.9	<0.001
(years)	standard deviation	9.9	9.4	8.8	9.5	

**Table 3:** List of free text answers by psychiatrists, psychologists, psychotherapists, and psychiatrists/psychologists in training regarding recommended diets for individuals with psychiatric disorders.

Free-Text Answers from Mental Health Professionals Regarding Recommended Diets for Individuals with Psychiatric Disorders	Description
“Alkaline diet/ Base-fasting”	Only foods that have a supposed alkaline metabolism are allowed for a preset period of time
“Blue Zone diet”	Low meat intake, high fiber, and minimally processed foods, originating from so-called ‘*Blue zones*’ described as being healthiest communities worldwide [26]
“Chrononutrition”	Nutrition considering the circadian system [27]
“Clean 9”	Diet using a supplement regime
“Dietary advices based on macrobiotic diet”	A basic nutritional scheme which is individualized depending on sex, age, level of activity, indivudal needs, and environment [28]
“Elimination reintroduction trials”	Individuals forgo certain foods or ingredients to find out if they have a negative effect on them
“F. X. Mayr Kur”	Detoxifying diet originally with bread rolls and milk [29]
“fasting”	Willful refrainment from eating
“FDH (=“Friss die Hälfte”) diet”	German phrase saying “eat half” (of what you normally would eat)
“FODMAP”	Restriction of rapidly fermentable, short-chain carbohydrates for patients with functional gut symptoms [30]
“Intermittent fasting”	Alternating time periods of regular food intake and fasting
“Metabolic balance diet”	Nutrition program aiming to change lifestyle permanently through individualized nutrition plans taking releant blood paramters into account for laboratory support [31]
“MIND”	Mediterranean DASH (Dietary Approaches to Stop Hypertension) Intervention for Neurodegenerative Delay [32]
“Nutrition according to the five elements”	Nutrional approach arised from Traditional Chinese Medicine (TCM). Local foods are categorized from an energetic pont of view [33]
“Sleep diet by Dr. Pape”	Diet concentrating on eating the right thing at the right time and take longer breaks between meals, in addition to an active everyday life and a lot of sleep [34]
“TCM (Traditional Chinese Medicine)”	TCM aims to achieve harmony and balance in ones body through food. It has its own internal logic and concepts [35]
“Traffic light system diet”	Foods are labeled in different groups according to their amount of health-relevant nutrients
in general	“balanced, diversified”
	“healthy”
	“unprocessed”
	“seasonal”, “regional”
	“reasonable with regard to carbs”
	
effects	“deacidifying”
	“warming dishes”
	“sleep promoting”
	“acid -base balance”
high/rich in	“whole foods”
	“(plant based) fresh fruit and vegetables”
	“nuts”
	“fiber”
	“omegas”
	“proteins”
	“antioxidants”
inclusion of	“fatty fish”
	“probiotic food such as fermented cabbage”
	“serotonin rich food”
free of or reduction of	“gluten”
	“casein”
	“caffeine/soft drings with caffeine”
	“wheat”
	“dairy”
	“allergenic foods”
	“meat”
	“sugar/sweets”
	“calories”
	“portion quantity”
	“instant meals”
	“conservats”
	“cholesterol”
	“salt”
	“fat”
	“sodium”
	“sodium-glutamate”
	“alcoholic foods”

**Table 4:** List of answers in a free text field regarding dietary specifications given by psychiatrists, psychologists, psychotherapists, and psychiatrists/psychologists in training.

Depending on Time	
“regular nutritional intake”	
“intermittent fasting”	
“no stimulating foods in the evening”	
“two meals a day”	
**depending on the process of the intake**	
“mindful eating”	
“awareness of nutrition”	
“no eating for stress relief”	
**adapted to individual requirements**	
diabetes	“diabetes diet”
eating disorders malnourished obesity	“diet plans, main and in-between meals” “fortification” “hypocaloric diet”
cachexia	“high calorie intake”
hyponatremia	“salty nutrition”
cancer	“depending on specific cancer”
serotonin side effects	“avoiding foods with tryptophan if suspected serotonin side effects are present”
Morbus Wilson	“low copper in nutrition in case of Morbus Wilson”
Geriatric patients	“I recommend old people having difficulties with eating or are anxious about it especially eating what gives them pleasure”
Monoamidoxidase (MAO-) inhibitors	“diet low in tyramine when thereis intake of MAO-inhibitors”
**Other answers**	
Referral to colleagues	“I refer to a Nutritionist” “I refer to Dietologists” “I feel not qualified to give advice”
Other lifestyle interventions	“I recommend to stay slim without diet”
	“I recommend evidence-based interventions”
	“not necessarily through diet” “sports” “ban of illegal drugs”

**Table 5:** List of answers in a free text field regarding previous recommendation of any other supplements which were not listed in the options of the questionnaire.

Free-Text Answers from Mental Health Professionals Regarding Recommended Supplements and Foods for Psychiatric Disorders	[Linnean Name], Specifications, and Supposed (Psychiatric) Effects of Supplements * Named by Mental Health Professionals
“5-Hydroxytryptophane (5-HTP)”	Naturally occurring amino acid, chemical precursor of serotonin, used as a nonpharmacological treatment for depression [36]
“Adaptogenic herbs”	Substances used in traditional and herbal medicine with the aim of stabilization and promoting adaptation to environmental factors [37]
“Albumin”	Family of globular proteins, found in blood plasma
“Alkalising supplements”	For example, sodium bicarbonate
“Aloe vera juice”	Juice of [Aloe barbadensis]
“Amino acids (several)/Protein powder”	Precursor to neurotransmitters (tryptophane, tyrosine)
“Ashwagandha”	[Withania somnifera], herb with gamma-aminobutyric acid (GABA-)ergic properties used in traditional medicine to reduce stress and enhance wellbeing [38,39]
“Astaxanthine”	Carotenoid with antioxidant and anti-inflammatory properties, produced by several freshwater and marine microorganisms, including bacteria, yeast, fungi, and microalgae [40]
“Beta-glucan”	Sugars found in cell walls of bacteria, fungi, yeasts, algae, lichens, and plants (barley, oats) [41]
“Bitter substances”	Substances found in vegetables and spices (e.g., radicchio, chicory, endive, cardamom, ginger) used to treat digestive issues in traditional medicine systems
“Calcium”	Essential element needed in large quantities, acting as an electrolyte with important functions in nerve conduction and building of bone mass
“Cannabidiol (CBD)”	Phytocannabinoid, traditionally used for treatment of anxiety, cognition, movement disorders, and pain [42]
“Chamomile tea”	Tea made from dried flowers of [Chamaemelum nobile]; traditionally used as a supplementary approach to treat sleep problems and depression [43]
“Cherries”	[Prunus cerasus], source of polyphenols and vitamin C with anti-oxidant and anti-inflammatory properties [44,45]
“Chia seeds”	Seeds of [Salvia hispanica], novel food, under preliminary research for potential effects on health [46]
“Chlorella”	[Chlorella species]; single-celled green algae, consumed as a health supplement primarily in the United States and in Japan
“Choline”	Essential nutrient for humans needed for the production of the neurotransmitter acetylcholine; dietary sources of choline and choline phospholipids include egg yolk, wheat germ, and meats, especially organ meats, such as beef liver [47]
“Chromium”	Chemical element, used as a dietary supplement, showing decreases of the sensitivity of 5-HT2A receptors [48]
“Coenzyme Q10”	Ubichinone, a coenzyme present in all cells, mainly in mitochondria, as an element of the electron transport chain [49]
“Copper”	Chemical element, with high serum levels in Wilson’s disease [50]
“Epigallocatechin gallate (EGCG)”	Abundant catechin in green tea; polyphenol, found to enhance sleep [51]
“Evening primrose oil”	[Oenothera], contains gamma-linolenic acid
“Feverfew”	[Tanacetum parthenium], medicinal herb, traditionally used for the prevention of migraine [52]
“Ginkgo”	[Ginkgo biloba], traditionally used alone or as an add-on therapy, in the treatment of mild cognitive impairment and dementia [53]
“Glycine”	Amino acid, inhibitory neurotransmitter in the central nervous system, required co-agonist along with glutamate for N-methyl-D-aspartate (NMDA) receptors [54]
“Green tea”	Infusion prepared from [Camellia sinensis], containing L-theanine, polyphenols, and polyphenol metabolites, used historically in medicine with conflicting results for psychiatric disorders [55,56]
“Griffonia”	[Griffonia simplicifolia]; a tropical plant native to West Africa, rich in 5-hydroxy-l-tryptophan (5-HTP), a precursor in the synthesis of serotonin (5-HT), traditionally used for the treatment of depression [57]
“Herbal medicines”	Traditional plant-derived medicines, often given in combinations
“Herbal mixture (seven herbs from Heidelberg: anise, cumin, fennel, wormwood, yarrow, burnet, and juniper)”	Traditional herbal mixture used in Germany (Heidelberg) for the treatment of digestive issues
“Huperzine”	Alkaloid compound found in [Huperzia serrata], a traditional Chinese medicine supplement. Huperzine has strong acetylcholine inhibiting properties and is used as an over the counter supplement for neurological disorders such as Alzheimer’s disease [58,59]
“Inositol”	Carbocyclic sugar, abundant in the brain and important for cell signal transduction in response to a variety of hormones, neurotransmitters, and growth factors, as well as osmoregulation; some studies investigated inositol for panic disorders and obsessive compulsive disorders [60,61]
“Iodine”	Chemical element; used to treat iodine-deficiency or thyreotoxicosis
“Kudzu/Japanese arrowroot”	[Pueraria montana] trailing perennial vines native to East Asia; a food supplement traditionally recommended for the treatment of alcohol abuse and dependence [62,63]
“L-Arginine”	Amino acid that is used in the biosynthesis of proteins with possible roles in atherosclerosis, redox stress and the inflammatory process, regulation of synaptic plasticity and neurogenesis, and modulation of glucose metabolism and insulin activity [64,65]
“L-Aspartate”	Amino acid that is used in the biosynthesis of proteins
“Lavender oil”	Oil derived from [Lavandula], herbal oil traditionally used for anxiety and sleep disturbances [66,67]
“Lecithin”	Group of yellow-brownish fatty substances occurring in animal and plant tissues, used as a dietary supplement for dementia [68]
“Lemon balm”	[Melissa officinalis]; used as a sleep-aid in traditional medicine; anxiolytic effects on mood, cognition, and memory have been shown in clinical trials. AChE inhibitory activity, stimulation of the acetylcholine and GABA-A receptors and matrix metallo proteinase-2 are potential mechanisms of action [69,70]
“L-Lysine”	Essential amino-acid in humans; found to reduce positive symptoms in schizophrenia in small pilot trials; reduced anxiety and improved stress response [71,72]
“L-Methylfolate”	Primary biologically active form of folate; used as an adjunctive antidepressant in major depressive disorder [73] and as a therapy for negative symptoms in schizophrenia [74]
“L-Ornithine”	Non-proteinogenic amino acid, which plays a role in the urea cycle [75]
“L-Theanine”	Amino acid analogue of the proteinogenic amino acids L-glutamate and L-glutamine, constituent of green tea;
“L-Tryptophan”	Amino acid that is used in the biosynthesis of proteins, which is converted into 5-hydroxytryptophan (5-HTP), which is then converted into serotonin and melatonin; dietary supplement used as an antidepressant, anxiolytic, and sleep aid with limited evidence for depression [76]
“Melatonin”	Hormone of the pineal gland, which regulates the sleep–wake cycle with antioxidant properties; improves sleep and has anti-depressant and anti-anxiety effects [77,78]
“Methylsulfonylmethane (MSM)”	Organosulfur compound; used in alternative medicine which crosses the blood–brain barrier with no known medical benefits for psychiatric disorders [79,80]
“Mineral tablets”	Common constituent of dietary supplements
“Mint tea/Peppermint”	Herbal infusion of [Mentha piperita, Mentha spicata]; used in traditional medicine for irritable bowel syndrome (IBS) symptoms; limited human studies, no clinical trials for psychiatric indications [81]
“Multivitamins”	A supplement containing a range of vitamins and/or dietary minerals.
“Passion flower”	[Passiflora L.], traditionally used as a sedative and anxiolytic [82]
“Phosphorylethanolamine”	Ethanolamine derivative; found to have specific effects on mitochondrial function [83]
“Plum juice”	[Prunus spec.] traditionally used as a dietary laxative; has anti-oxidant and anti-inflammatory properties [84]
“Polyphenols”	Naturally occurring organic compounds characterized by multiples of phenol units, used for improving cognitive performance and symptoms of depression [85,86]
“Potassium”	Given as potassium chloride used in the treatment of hypokalemia
“Prebiotics (several)”	Non-digestible fiber, promoting growth of microorganisms; did not differ from placebo in trials for depression and anxiety in a recent meta-analysis [87], use of prebiotics still lacks sufficiently robust evidence for psychiatric disorders [88]
“Propolis”	Mixture of bees wax and saliva produced by honey bees; used in traditional medicine
“Pycnogenol”	Chemical compound found in the bark of European pine trees/[Pinus pinaster]; nutritional supplement used in alternative medicine for the treatment of attention deficit hyperactivity disorder (ADHD);
“Red clover”	[Trifolium pratense], a herb containing phytoestrogens, shown to increase cognitive function in postmenopausal women [89]
“Rose root”	[Rhodiola rosea], adaptogen traditionally used for the reduction of stress-related syndromes, such as fatigue and burnout [90]
“S-Adenosyl-L-Methionine (SAMe)”	Co-substrate involved in methyl group transfers, transsulfuration, and aminopropylation, used as an add-on therapy for depression [91]
“Saffron”	[Crocus sativus], used for symptoms of depression and anxiety [92,93]
“Salt (Sodiumchloride, NaCl)”	Used as saline solution for a number of indications in clinical medicine
“Seaweed oil”	Oil from macroalgae rich in phytosterols, carotenoids, and polysaccharides; extracts used in diet pills to lose weight; compounds cross the blood–brain barrier and exert neuro-protective functions [94,95]
“Silibinum”	[Silybum marianum] active compound from the milk thistle; traditionally used for hepatic disorders; plant-based intervention used for obsessive compulsive disorder and anxiety disorder [96]
“Sip foods”	Used as additional calorie sources in the treatment of anorexia nervosa
“Soy products”	Products made from soybeans [Glycine max]; used for improvement of cognitive function in adults [97]
“St. Johns Wort”	[Hypericum perforatum], used for mild to moderate major depression [98]
“Tumeric, Curcumin”	[Curcuma longa], spice frequently used in Asian countries with anti-inflammatory and anti-oxidant properties with effects on depressive and anxiety symptoms [99]
“Tyrosine”	Amino acid, precursor of dopamine and noradrenaline, thyroid hormones, and melanin
“Valerian”	[Valeriana officinalis], GABA-modulating phytochemical traditionally used as an anxiolytic [100]
“Vitamin B1”	Thiamine, used for the prevention of Wernicke-Korsakoff-syndrome in alcohol dependency disorders [101]
“Vitamin B3”	Vitamin family that includes three forms or vitamers: nicotinamide (niacinamide), niacin (nicotinic acid), and nicotinamide riboside; deficiencies cause pellagra (fatigue, loss of appetite, abdominal pain); co-factor in serotonin-synthesis [102]
“Vitamin B-Complex”	Complex of water-soluble B vitamins in food supplements
“Vitamin C”	Co-factor in serotonin-synthesis, with deficiencies linked to depression and cognitive impairment [103]
“Vitamin K2”	Menachinone, one of three types of vitamin K with protective effects on bone mineral density. May be beneficial for prevention of bone loss in patients with anorexia nervosa [104]
“Wild yams”	[Dioscorea villosa]; traditionally used root containing phytoestrogens
“Zeolite”	Aluminosilicate minerals used as adsorbents [105,106]

* In the case of someone recommending a specific brand name, the active ingredient is given in the table. In instances where a herbal supplement was mentioned, the Linnean classification of the herb is given in brackets.

**Table 6:** List of additional comments by survey participants when asked about supplements in psychiatric and psychological care.

**Free Text Answers from Psychiatrists**
“I refer to a dietitian”
“I recommend monitoring the blood sugar level”
“If a patient had a problem of malabsorption, bad digestion, cancer, operated bypass sleeve, etc., I recommend protein with specific check-ups and specific products and laboratory results (before I worked as a doctor in internal medicine)”
“I warn my patients to be cautious for interactions when they are taking supplements together with psychopharmacological medication”
**Free Text Answers from Psychologists and Psychotherapists**
“I recommended to see a medical doctor when a patient asked me to recommend a supplement”
“I send my clients to see a doctor if they ask me about nutrition”
“I ask my patients to go to the doctor and check up and take some proper supplements. Or telling them to have some special fruits and vegetables to get vitamin B, iron, omega 3 etc.”
“Any discussion I have is by asking them to go to a general practitioner (GP) to discuss above”
“I recommended to see a naturopath”
“I recommended the patient to inform them about supplements”
“Do not see it as my role as a psychologist”

## Data Availability

Data is contained within the article or supplementary material. The data presented in this study are available in Supplementary File 2.

## Supplementary Materials

The following are available online at https://www.mdpi.com/2072-6643/13/3/822/s1, Supplementary File S1: Questions of the survey. Supplementary File S2: Survey answers.
