# Supplementary material for: ‘An Apple a Day’?: Psychiatrists, Psychologists and Psychotherapists Report Poor Literacy for Nutritional Medicine: International Survey Spanning 52 Countries
Source: Nutrients. 2021 Mar 2;13(3):822. doi: 10.3390/nu13030822 (PMC8000813; doi:10.3390/nu13030822)
Supplement: Supplementary file 1 [file nutrients-13-00822-s001.zip › nutrients-1075657-supplmentary files/nutrients-1075657_Supplementary-File1.pdf]

Survey Nutritional Psychiatry - How much do we know about nutrition in psychiatric and psychological care? / Umfrage Nutritional Psychiatry - Wie viel wissen wir über Ernährung in der psychiatrischen und psychologischen Versorgung?

Meaning of different shapes: O only one answer could be given， □ multiple answers could be given.

Daily nutritional intake is a central component of physical fitness. Nutritional status is increasingly being recognised as fundamental for the prevention of psychiatric disorders and as part of an holistic treatment plan for psychiatric illness. However, the general approach to patient nutrition varies among professionals across the world.

This survey aims to evaluate current experience and practice of psychiatrists and psychologists from all over the world regarding the area of 'Nutritional Psychiatry'.

The survey will take approximately 10-15 minutes.

The survey is anonymous and there is no possibility of personal identification. The submitted data cannot be corrected or withdrawn. There is no registration process for the survey and the data cannot be traced back to you.

Thank you for your participation!

/

Tägliche Ernährungsanforderungen werden international als ein zentraler Aspekt der körperlichen Gesundheit angesehen. Des Weiteren spielt die Aufklärung betreffend Ernährung und Nahrungsergänzungsmittel in der modernen Psychiatrie und Psychologie eine immer größere Rolle in der PatientInnenenbehandlung und primären Prävention von psychiatrischen Erkrankungen. Die allgemeine Herangehensweise an das Thema Ernährung variiert allerdings zwischen den SpezialistInnen in verschiedenen Ländern.

In dieser Studie sollen derzeitige Erfahrungen und Praktiken von PsychiaterInnen und PsychologInnen aus der ganzen Welt bezüglich Nutritional Psychiatry evaluiert werden.

Diese Umfrage wird ca. 10-15 Minuten in Anspruch nehmen.

Diese Umfrage ist anonym. Es ist keine persönliche Identifikation möglich, die eingegebenen Daten können somit nicht korrigiert oder widerrufen werden. Eine Registrierung zum Ausfüllen des Fragebogens ist nicht erforderlich. Die erhobenen Daten können nicht auf Ihre Person zurückverfolgt werden.

Vielen Dank für Ihre Teilnahme!

Supplementary File 1- Online Questionnaire

Consent / Einverständniserklärung

Dear participant!

We warmly invite you to take part in this survey.

Surveys are necessary to collect reliable new information for medical research. However, an essential requirement for the accomplishment of a survey is your written agreement for participation. Please read the following agreement thoroughly.

By ticking the box "I agree" below, you give your consent to take part in the survey.

Participation in this survey is voluntary. You can stop taking part in this survey at any time.

The data we collect through this survey may be used for scientific publications. This is an anonymous questionnaire. This means that a connection between the results and an individual person cannot be made.

Data will not be transferred to third parties.

If you have any questions regarding the use of data in this survey, please contact the doctor responsible for this survey (sabrina.moerkl@medunigraz.at). She can forward your questions to persons which are responsible for data protection.

Data protection officer: datenschutz@medunigraz.at

/

Sehr geehrte Teilnehmerin, sehr geehrter Teilnehmer!
Wir laden Sie herzlich ein, an dieser Umfrage teilzunehmen.

Durch das Ankreuzen des unten stehenden Kästchens "Ich stimme zu", erklären Sie sich mit den folgenden Bedingungen einverstanden:
Die Teilnahme an dieser Umfrage erfolgt freiwillig. Sie können jederzeit die Teilnahme an der Umfrage beenden.

Umfragen sind notwendig, um verlässliche neue medizinische Forschungsergebnisse zu gewinnen. Unverzichtbare Voraussetzung für die Durchführung einer Umfrage ist jedoch, dass Sie Ihr Einverständnis zur Teilnahme an dieser Umfrage schriftlich erklären. Bitte lesen Sie den folgenden Text sorgfältig durch.

Bei Fragen können Sie sich an die Studienverantwortliche DDr. Sabrina Mörkl (sabrina.moerkl@medunigraz.at) wenden.
Bitte beginnen Sie nur mit dem Ausfüllen des Fragebogens, wenn Sie einverstanden sind an dieser Umfrage teilzunehmen. Durch Anhaken des Kästchens unter dieser Erklärung bestätigen Sie, dass Sie damit einverstanden sind teilzunehmen.

Die Daten, die im Rahmen dieser Befragung erhoben werden, dürfen für wissenschaftliche Publikationen verwendet werden. Es handelt sich zudem um einen anonymisierten Fragebogen. Dies

bedeutet, dass kein Zusammenhang zwischen den Ergebnissen und Ihrer Person hergestellt werden kann. Daten werden nicht an Dritte weitergegeben.

Falls Sie Fragen zum Umgang mit Ihren Daten in dieser Umfrage haben, wenden Sie sich zunächst an Ihre Prüfärztin (sabrina.moerkl@medunigraz.at). Diese kann Ihr Anliegen ggf. an die Personen, welche für den Datenschutz verantwortlich sind, weiterleiten.
Datenschutzbeauftragte/r des Prüfzentrums, Krankenhausträger: datenschutz@medunigraz.at

Do you agree with the terms listed above? / Sind Sie mit den oben genannten Bedingungen einverstanden?

- I agree / Ich stimme zu
- I disagree / Ich stimme nicht zu

Survey - Demographical data / Umfrage - Demographische Daten

Age / Alter
How old are you? / Wie alt sind Sie?

Sex / Biologisches Geschlecht

- Male / Männlich
- Female / Weiblich

Country of occupation / Land des Beschäftigungsverhältnisses
Which country do you currently work in? / In welchem Land haben Sie derzeit eine Beschäftigung?

City of occupation / Stadt des Beschäftigungsverhältnisses
Which city do you currently work in? / In welcher Stadt haben Sie derzeit eine Beschätigung?

Institution / Einrichtung
What kind of institution do you currently work at? / In welcher Einrichtung sind Sie derzeit tätig?

- Hospital / Krankenhaus
- Mental health outpatient department / Psychosozialer Dienst/psychiatrische Ambulanz
- Day clinic / Tagesklinik
- Rehabilitation center / Rehabilitationszentrum
- Private practice / eigene Praxis bzw. Ordination
- Other /Andere

If you have chosen "other" in the question before, please shortly describe the institution you work at. / Wenn Sie in der vorangegangenen Frage "andere" gewählt haben, beschreiben Sie bitte kurz die Einrichtung in der Sie tätig sind!

Subdivision / Abteilung
What subdivision do you mainly engage in? / In welcher Abteilung sind Sie primär tätig?
Inpatient care / Stationäre Behandlung

- ☐ Outpatient care / Ambulante Behandlung
- ☐ Day care hospital / Tagesklinik
- ☐ Psychotherapy outpatient care / Ambulante psychotherapeutische Behandlung
- ☐ Private practice / Eigene Praxis bzw Ordination
- ☐ University and education / Universität und Lehre
- ☐ In training / In Ausbildung
- ☐ General practitioner / Allgemeinmediziner

What kind of qualification(s) do you have? / Welche Qualifikation(en) haben Sie?

- ☐ Consultant psychiatrist / Facharzt oder Fachärztin für Psychiatrie
- ☐ Psychologist / PsychologIn
- ☐ Psychotherapist / PsychotherapeutIn
- ☐ Psychiatrist or psychologist in training / Zusätzliche Ausbildung
- ☐ GP general practitioner / Allgemeinmediziner
- ☐ Consultant - other specialty / Facharzt oder Fachärztin andere Disziplin

If you have chosen "consultant- other specialty"- what field are you specialized in? / Wenn Sie bei der vorangegangenen Frage "Facharzt oder Fachärztin- andere Disziplin" gewählt haben- in welcher Disziplin haben Sie sich spezialisiert?

Are you specialized in a certain field? / Haben Sie sich auf ein bestimmtes Gebiet spezialisiert?

- o General adult psychiatry or psychology / Psychiatrie oder Psychologie für Erwachsene
- o Child and adolescent psychiatry or psychology / Kinder- und Jugendpsychiatrie oder Psychologie
- o Psychogeriatrics / Psychogeriatrie
- o Addiction Medicine / Suchtmedizin
- o Forensic psychiatry or psychology / Forensische Psychiatrie oder Psychologie
- o Neuropsychiatry or neureopsychology / Neuropsychiatrie oder Neuropsychologie
- o Psychosomatics / Psychosomatik
- o Other / Andere
- o None / Keine

If you answered "other" in the question before, please specify which field you are specialized in. / Wenn Sie in der vorangegangenen Frage "Andere" geantwortet haben, bitte spezifizieren Sie in welchem Gebiet Sie sich spezialisiert haben.

Working experience in psychiatry or psychology / Arbeitserfahrung in Psychiatrie oder Psychologie

How many years have you been practicing psychiatry/psychology? (including training period and residency (excluding university education)) / Wie viele Jahre haben Sie in der Psychiatrie oder Psychologie gearbeitet (inklusive Ausbildung, Facharztausbildung, Ausbildung zur klin. Psychologin (exkl. Studium))?

Year of postgraduate education / Postgraduelles Ausbildungsjahr

If you are in psychiatric or psychological training: which year of your postgraduate studies are you currently in? / Falls Sie sich in psychiatrischer oder psychologischer Ausbildung befinden: in welchem postgraduellen Ausbildungsjahr sind Sie derzeit?

- o 1
- o 2
- o 3
- o 4
- o 5
- o 6
- o > 6
- o I have completed my studies / Ich habe meine Ausbildung abgeschlossen

Country specific period of postgraduate studies / Studiendauer der postgraduellen Ausbildung in Ihrem Land
Studying full time, how many years does it usually take to finish your postgraduate studies enabling you to work with patients independently as a psychiatrist or psychologist in your country? / Wie lange dauert die postgraduelle Ausbildung bei einem Vollzeitstudium in Jahren, um mit PatientInnen eigenständig als PsychiaterIn oder PsychologIn in Ihrem Land arbeiten zu dürfen?

- o 3 or less / 3 oder weniger
- o 4
- o 5
- o 6
- o 7
- o 8 or more / 8 oder mehr

Nutritional Psychiatry - Knowledge / Wissen

Current knowledge and its acquirement / Aktueller Wissensstand und dessen Aneignung

Please note: "patients" always refers to persons with a suspected or diagnosed mental disorder / Bitte beachten Sie: "PatientInnen" bezieht sich immer auf Personen, bei denen der Verdacht oder die Diagnose einer psychischen Störung vorliegt.

Did you ever use nutritional approaches for the treatment of your patients? If yes, for which indications (mainly)? / Haben Sie jemals Ernährungsmaßnahmen in der Behandlung von PatientInnen eingesetzt? Wenn ja, bei welchen Indikationen haben Sie diese (hauptsächlich) eingesetzt?

- ☐ Affective disorders / Affektive Störungen
- ☐ Anxiety disorders / Angsterkrankungen
- ☐ Psychotic disorders / Psychotische Erkrankungen
- ☐ Obsessive compulsive disorder / Zwangsstörung
- ☐ Eating disorders / Esstörungen
- ☐ Others / Andere
- ☐ No, never / Nein, niemals

From 1 to 10 - In your opinion, how important is nutritional care in the context of mental disorders? / Von 1 bis 10 - Wie wichtig ist Ihrer Meinung nach ernährungsbezogene Versorgung im Kontext von psychischen Störungen?

	1	2	3	4	5	6	7	8	9	10	
Very low importance / Gar nicht wichtig	O	O	O	O	O	O	O	O	O	O	Very high importance / Sehr wichtig

Supplementary File 1- Online Questionnaire

From 1 to 10 - In your institution, how important is discussing nutrition with your patients? / Von 1 bis 10 - Wie wichtig ist es in Ihrer Institution, mit PatientInnen deren Ernährung zu besprechen?

	1	2	3	4	5	6	7	8	9	10	
Very low importance / Gar nicht wichtig	O	O	O	O	O	O	O	O	O	O	Very high importance / Sehr wichtig

If you are a psychologist: Was Nutritional Psychiatry (nutritional approaches for mental disorders) discussed in your postgraduate studies? / Für PsychologInnen: Wurde im Rahmen Ihrer postgraduellen Ausbildung das Thema Nutritional Psychiatry (Ernährung bei psychischen Erkrankungen) durchgenommen?

- Yes, obligatory / Ja, verpflichtend
- Yes, but only because I attended electives about this topic / Ja, aber nur weil ich Wahlfächer zu diesem Thema belegt habe
- No, but I had some training during my studies of psychology / Nein, aber im Psychologiestudium wurde das Thema behandelt
- No/Nein

If you are a psychiatrist: Did you have specific training in nutritional care of patients during your psychiatric residency? / Für PsychiaterInnen: Hat es im Rahmen der Facharztausbildung spezielle Lehre zum Thema Nutritional Psychiatry (Ernährung bei psychischen Erkrankungen) gegeben?

- Yes, obligatory / Ja, verpflichtend
- Yes, but only because I attended electives about this topic / Ja, aber nur weil ich Wahlfächer zu diesem Thema belegt habe
- No / Nein
- No, but I had some training during my diploma programme of humane medicine / Nein, aber im Studium Humanmedizin wurde das Thema behandelt

Are you aware of any courses that teach nutrition for prevention and treatment of psychiatric disorders in your country or at your institution? / Sind Ihnen Kurse bekannt, in denen Ernährung zur Prävention und Behandlung von psychischen Erkrankungen in Ihrem Land oder an Ihrer Institution gelehrt werden?

- Yes / Ja
- No / Nein

If yes, did you attend any specific training in nutritional care of patients while working as a psychiatrist or psychologist? / Wenn ja, haben Sie in Ihrer Zeit als PsychiaterIn oder PsychologIn bisher an einem speziellen Kurs über Ernährung von PatientInnen teilgenommen?

- Yes / Ja
- No / Nein

From 1 to 10 - How would you rate your current knowledge regarding Nutritional Psychiatry? / Von 1 bis 10 - Wie würden Sie Ihren aktuellen Wissensstand über Nutritional Psychiatry einstufen?

	1	2	3	4	5	6	7	8	9	10	
Very low / Sehr niedrig	O	O	O	O	O	O	O	O	O	O	Very high / Sehr hoch

From 1 to 10 - How would you rate the necessity for further research in Nutritional Psychiatry? / Von 1 bis 10 - Wie würden Sie die Notwendigkeit für weitere Forschung im Bereich Nutritional Psychiatry einstufen?

No necessity / Keine Notwendigkeit	1	2	3	4	5	6	7	8	9	10	High necessity / Hohe Notwendigkeit
	O	O	O	O	O	O	O	O	O	O	

From 1 to 10 - How would you rate the necessity for psychiatrists and psychologists to further train in Nutritional Psychiatry? / Von 1 bis 10 - Wie würden Sie die Notwendigkeit für zusätzliche Ausbildungen/Fortbildungen in Nutritional Psychiatry für PsychiaterInnen und PsychologInnen einstufen?

No necessity / Keine Notwendigkeit	1	2	3	4	5	6	7	8	9	10	High necessity / Hohe Notwendigkeit
	O	O	O	O	O	O	O	O	O	O	

From 1 to 10 - Do you think you could improve the quality and outcome of your work by (further) training in Nutritional Psychiatry? / Von 1 bis 10- Denken Sie, dass Sie die Qualität und das Resultat Ihrer Arbeit durch (weiteres) Training in Nutritional Psychiatry verbessern könnten?

Not at all / Überhaupt nicht	1	2	3	4	5	6	7	8	9	10	Yes, definitely / Ja, definitiv
	O	O	O	O	O	O	O	O	O	O	

Would you personally like to expand your knowledge about Nutritional Psychiatry? / Würden Sie persönlich gerne Ihr Wissen über Nutritional Psychiatry ausbauen?

- o Yes / Ja
- o No / Nein

If yes, in which setting would you like to improve your knowledge? / Wenn Ja, in welchem Rahmen würden Sie gerne Ihr Wissen erweitern?

- ☐ Interdisciplinary meetings / Interdisziplinäre Sitzungen
- ☐ Training during studies / Ausbildungen und Fortbildungen während der Ausbildung
- ☐ Master studies / Master-Studium
- ☐ PhD project-thesis / PhD Projekt-Dissertation
- ☐ Congresses and events / Kongresse und Veranstaltungen
- ☐ Scientific Journals / Wissenschaftliche Zeitschriften
- ☐ Journal club / Journal Club

Nutritional Psychiatry - Treatment / Behandlung

Current treatment practices / Aktuelle Behandlungsmethoden

Please note: "patients" always refers to patients suspected or diagnosed with a mental disorder / Bitte beachten Sie: "PatientInnen" bezieht sich immer auf PatientInnen, bei denen der Verdacht oder die Diagnose einer psychischen Störung vorliegt.

From 1 to 10 – What do you think about the current nutritional status of your countries‘ population? / Von 1 bis 10 – Wie beurteilen Sie die derzeitige Ernährungsweise der Menschen in Ihrem Land?

Low nutritional status /Geringe Qualität	1	2	3	4	5	6	7	8	9	10	High nutritional status/ Hohe Qualität
	O	O	O	O	O	O	O	O	O	O	

Supplementary File 1- Online Questionnaire

From 1 to 10 - What do you think about the current nutritional status of people with mental disorders in your country?/ Von 1 bis 10- Wie beurteilen Sie die derzeitige Ernährungsweise von Menschen mit psychischen Erkrankungen in Ihrem Land?

	1	2	3	4	5	6	7	8	9	10	
Low nutritional status /Geringe Qualität	O	O	O	O	O	O	O	O	O	O	High nutritional status/ Hohe Qualität

From 1 to 10 - How would you rate the average nutritional value of the food served in hospitals in your country? / Von 1 bis 10 - Wie schätzen Sie die durchschnittliche Qualität der Nahrung in den Krankenhäusern in Ihrem Land ein?

	1	2	3	4	5	6	7	8	9	10	
Low nutritional value / Geringe Qualität	O	O	O	O	O	O	O	O	O	O	High nutritional value / Hohe Qualität

From 1 to 10 - How often do you usually talk to your patients about their diet? / Von 1 bis 10 - Wie häufig reden Sie üblicherweise mit Ihren PatientInnen über deren Ernährung?

	1	2	3	4	5	6	7	8	9	10	
Never / Nie	O	O	O	O	O	O	O	O	O	O	Always / Immer

Is there regular screening for metabolic disorders for psychiatric patients in your country? / Gibt es ein Screening für metabolische Erkrankungen bei PatientInnen mit psychiatrischen Erkrankungen in Ihrem Land?

- Ja / Yes
- Nein / No
- Ich weiß es nicht / I do not know

Do you treat your patients using nutritional interventions for psychiatric disorders? / Behandeln Sie Ihre PatientInnen auch mithilfe von ernährungsbezogenen Interventionen bei psychiatrischen Erkrankungen?

- Sometimes / Manchmal
- Always / Immer
- Hardly ever / Selten
- Most of the time / Meistens
- Never / Nie

Do you use nutritional interventions for the prevention of somatic comorbidities? / Verwenden Sie ernährungsbezogene Interventionen zur Prävention somatischer Komorbiditäten?

- Always / Immer
- Most of the time / Meistens
- Sometimes / Manchmal
- Hardly ever / Selten
- Never / Nie

Do you consider the individual nutrition of your patients when prescribing psychopharmacological therapy? / Berücksichtigen Sie die individuelle Ernährung Ihrer PatientInnen in Bezug auf die verschriebene psychopharmakologische Medikation?

- Always / Immer
- Most of the time / Meistens
- Sometimes / Manchmal
- Hardly ever / Selten
- Never / Nie

Supplements / Nahrungsergänzungsmittel: Did you ever recommend nutritional supplements to your patients? / Haben Sie Ihren PatientInnen je Nahrungsergänzungsmittel empfohlen?

- o Yes / Ja
- o No / Nein

If yes, which ones did you recommend? / Wenn ja, welche haben Sie empfohlen?

- ☐ Vitamin D
- ☐ Omega 3
- ☐ Vitamin A
- ☐ Vitamin E
- ☐ Selenium / Selen
- ☐ Zinc / Zink
- ☐ Magnesium
- ☐ Vitamin B6 / Vitamin B6
- ☐ Vitamin B12 / Vitamin B12
- ☐ Folic acid / Folsäure
- ☐ Iron / Eisen
- ☐ N-acetylcysteine / N-Acetylcystein
- ☐ Other / Andere

Did you recommend any other supplements which were not mentioned above? If yes, please name them. / Haben Sie weitere Nahrungsergänzungsmittel empfohlen, die oben nicht genannt wurden? Wenn ja, welche waren das?

Did you ever take supplements yourself? / Haben Sie selbst je Nahrungsergänzungsmittel eingenommen?

- o Yes / Ja
- o No / Nein

Probiotics / Probiotika: Did you ever recommend probiotics to your patients? / Haben Sie Ihren PatientInnen je Probiotika empfohlen?

- o Yes / Ja
- o No / Nein

Special diets / Spezielle Ernährungsformen: Did you ever recommend a special diet to your patients? / Haben Sie Ihren PatientInnen je eine spezielle Diät/Ernährungsform empfohlen?

- o Yes / Ja
- o No / Nein

If yes, which kind(s) of diet(s)? / Wenn ja, welche Ernährungsform(en)?

- ☐ Diet according to national dietary guidelines / Ernährungsform nach nationalen Empfehlungen
- ☐ Mediterranean diet / Mediterrane Diät
- ☐ Vegetarian diet / Vegetarische Ernährung
- ☐ Vegan diet / Vegane Ernährung
- ☐ Ketogenic diet / Ketogene Diät
- ☐ Low carb diet / Diät mit wenigen Kohlenhydraten "low carb"
- ☐ Glyx diet / Glyx Diät
- ☐ Other / Andere

If you chose the option "other" in the question before, which diet did you recommend? / Wenn Sie in der vorangegangenen Frage die Option "andere" gewählt haben- Welche Ernährungsform haben Sie empfohlen?

☐

Please specify the indications for recommending the diet(s): / Bitte geben Sie die Indikationen für die Empfehlung der Diät(en) an:

- ☐ Symptoms of depression / Depressive Symptomatik
- ☐ Symptoms of anxiety / Angstsymptomatik
- ☐ Psychosis / Psychosen
- ☐ Dementia / Demenz
- ☐ Eating disorders / Essstörungen
- ☐ Personality disorders / Persönlichkeitsstörungen
- ☐ ADHD / ADHS
- ☐ Obsessive compulsive disorder / Zwangsstörungen
- ☐ Obesity / Obesitas
- ☐ Metabolic comorbidities / Metabolische Komorbiditäten
- ☐ Prevention of adiposity and metabolic comorbidities / Vorbeugung von Adipositas und metabolischen Komorbiditäten
- ☐ Others

Have you ever started a diet yourself and proceeded for more than one month? / Haben Sie je selbst eine Diaet begonnen und fuer mehr als einen Monat durchgefuehrt?

- o Yes / Ja
- o No / Nein

Did you ever recommend lifestyle interventions to your patients? If yes, which ones? / Haben Sie ihren PatientInnen jemals Lebensstilveränderungen empfohlen? Wenn ja, welche?

- ☐ Hardly ever recommended lifestyle interventions / Ich habe fast nie Lebensstilinterventionen empfohlen
- ☐ Dietary coaching / Ernährungsberatung
- ☐ Cooking classes / Kochkurse
- ☐ Physical activity / Körperliche Aktivität
- ☐ Other / Andere

Food allergies, gluten-sensitivity or food intolerances / Nahrungsmittelallergien, Gluten-Sensitivität oder Nahrungsmittelintoleranzen

How often do you initiate a test for food allergies, gluten-sensitivity or food intolerances for your patients? / Wie häufig initiieren Sie einen Test auf Nahrungsmittelallergien, Gluten-Sensitivtät oder Nahrungsmittelintoleranzen bei Ihren PatientInnen?

- o Always / Immer
- o Most of the time / Meistens
- o Sometimes / Manchmal
- o Hardly ever / Selten
- o Never / Nie

Thank you for your participation! / Danke für Ihre Teilnahme!
